# Metabolomic markers of fatigue: Association between circulating metabolome and fatigue in women with chronic widespread pain

**DOI:** 10.1016/j.bbadis.2017.11.025

**Published:** 2018-02

**Authors:** Maxim B. Freidin, Helena R.R. Wells, Tilly Potter, Gregory Livshits, Cristina Menni, Frances M.K. Williams

**Affiliations:** a Department of Twin Research and Genetic Epidemiology, King's College London, London, UK; b Department of Anatomy and Anthropology, Sackler Faculty of Medicine, Tel Aviv University, Israel

**Keywords:** Fatigue, Chronic widespread pain (CWP), Metabolome, Eicosapentaenoate (EPA)

## Abstract

**Background:**

Fatigue is a sensation of unbearable tiredness that frequently accompanies chronic widespread musculoskeletal pain (CWP) and inflammatory joint disease. Its mechanisms are poorly understood and there is a lack of effective biomarkers for diagnosis and onset prediction. We studied the circulating metabolome in a population sample characterised for CWP to identify biomarkers showing specificity for fatigue.

**Material and methods:**

Untargeted metabolomic profiling was conducted on fasting plasma and serum samples of 1106 females with and without CWP from the TwinsUK cohort. Linear mixed-effects models accounting for covariates were used to determine relationships between fatigue and metabolites. Receiver operating curve (ROC)-analysis was used to determine predictive value of metabolites for fatigue.

**Results:**

While no association between fatigue and metabolites was identified in twins without CWP (n = 711), in participants with CWP (n = 395), levels of eicosapentaenoate (EPA) ω-3 fatty acid were significantly reduced in those with fatigue (β = − 0.452 ± 0.116; p = 1.2 × 10^− 4^). A significant association between fatigue and two other metabolites also emerged when BMI was excluded from the model: 3-carboxy-4-methyl-5-propyl-2-furanpropanoate (CMPF), and C-glycosyltryptophan (p = 1.5 × 10^− 4^ and p = 3.1 × 10^− 4^, respectively). ROC analysis has identified a combination of 15 circulating metabolites with good predictive potential for fatigue in CWP (AUC = 75%; 95% CI 69–80%).

**Conclusion:**

The results of this agnostic metabolomics screening show that fatigue is metabolically distinct from CWP, and is associated with a decrease in circulating levels of EPA. Our panel of circulating metabolites provides the starting point for a diagnostic test for fatigue in CWP.

## Highlights

• Fatigue is a debilitating condition of unknown aetiology associated with chronic pain.
• Metabolome provides an agnostic tool to reveal mechanisms of complex diseases.
• Decreased circulating levels of eicosapentoenoate (EPA) were associated with fatigue coupled with chronic pain.
• The set of 15 circulating metabolites provide a diagnostic tool for fatigue associated with chronic pain.

## 1 Introduction

Fatigue is a condition of debilitating tiredness, lethargy and lack of energy which manifests as a symptom of many different diseases and, more rarely, on its own (chronic fatigue syndrome, CFS, also known as myalgic encephalomyelitis, ME). The prevalence of fatigue may be as high as 50% in the general population, though in most cases it is transient and diminishes as the causal factor (e.g. viral infection) resolves [1], [2]. Fatigue is a characteristic symptom of many chronic rheumatic conditions such as rheumatoid arthritis (RA), system lupus erythematous (SLE), and fibromyalgia. Patients with SLE and RA report persistent fatigue in up to 80–90% cases and there is no clear evidence that it is related to disease activity [3], [4], [5]. Fatigue is also common in non-inflammatory conditions such as cancer and neurological disorders.

Fatigue is often associated with chronic pain; in rheumatic diseases both the presence and the intensity of these symptoms are well correlated [3], [4], [6], [7]. In patients with RA and SLE painful symptoms such as arthralgia are important contributing factors to fatigue severity; also, pain has been shown to be the strongest predictor of fatigue in many studies [4], [8], [9].

Fatigue often occurs in fibromyalgia, a non-inflammatory condition manifesting chronic widespread pain (CWP) accompanied by sleep disturbance [10]. Due to the strong co-morbidity of fibromyalgia and chronic fatigue some researchers debate they are a single syndrome caused by overlapping mechanisms [11]. This is supported by a genetic epidemiology study that showed strong genetic correlation between CWP and fatigue, suggestive of the presence of shared genes and molecular pathways [12]. On the other hand, other studies proposed that co-occurrence between fatigue and CWP is due to their share of common psychiatric component (anxiety and depression) [13] which also shares genetic determinants with CWP [12].

The metabolome comprises the sum of small molecule chemicals (amino acids, lipids, fatty acids, sugars, vitamins, etc.) detectable in a sample, usually serum, plasma or urine. It represents the higher end of phenotypic expression of the genome and is, therefore, much closer to the phenotype of interest than, say, protein expression. Metabolomic studies are increasingly successful in identifying mechanisms of complex diseases and may reveal new targets for therapy and provide diagnostic and prognostic tools [14], [15], [16].

We have recently demonstrated that individuals with CWP may present with an altered metabolic profile compared to healthy individuals [17]. It is also supported by the findings that the risk of CWP increases with higher body mass index (BMI) [18]. Furthermore, dietary risk factors which affect metabolite levels, including higher consumption of fats but lower consumption of fruit and vegetables, have been found in those with CWP [18].

There are limited metabolome studies in fatigue [19], [20], [21], [22], [23], [24], [25] and no studies have examined fatigue in the general population as opposed to a clinical sample. We investigated the circulating metabolome in a large sample of twins taken from the UK population. We were interested to determine whether there is a pattern of circulating metabolome specific for fatigue in people reporting CWP which might provide a diagnostic biomarker for this symptom.

## 2 Material and methods

### 2.1 Sample

Participants were a sample of MZ and DZ twins enlisted in the TwinsUK registry [26]. This is a bioresource which has been collected and maintained by the Department of Twin Research and Genetic Epidemiology at King's College London over the last 25 years. Ethical approval is available from the St Thomas' Hospital Research Ethics Committee and, at each visit, the participating twins provided fully informed consent for their biological specimens, clinical and demographic information to be used in molecular epidemiology studies. Many of the data including metabolome are available to internal and external researchers subject to approval by the Twin Research Executive Committee (http://www.twinsuk.ac.uk/data-access/submission-procedure/). Complete confidentiality was assured and the twins were unaware of any specific hypotheses.

An original sample for the current study comprised 4898 twins with known status of CWP established as described elsewhere [17]. The status of fatigue was assessed using three self-administered health questionnaires collected in 2000, 2002 and 2008 (Supplementary Table 1). Even though the definitions of fatigue using different time points (2000 vs 2002 and 2008) were not the same; the prevalence of fatigue using definitions from questionnaires of 2000 and 2008 were similar: 25.2% and 22.6%, respectively, and did not differ statistically (p = 0.494). Only two persons from 2002 were included in the final sample, both having no fatigue. Therefore, we analysed all the sample together to increase the sample size and statistical power. Collection of concomitant socio-demographic information and physical examination was carried out during twin visits or via self-administered questionnaires. Each twin completed the questionnaires without reference to co-twin and were unaware of the precise research hypothesis addressed in the study. Twins reporting inflammatory disease such as RA, SLE and inflammatory bowel disease were excluded from the study.

### 2.2 Metabolome

Non-targeted metabolite detection and quantification of 280 structurally named biochemicals was conducted by the metabolomics provider Metabolon, Inc. (Durham, USA) on fasting plasma or serum samples from the participants, as described previously [27]. Day median normalization followed by inverse transformation of ranks to normality was applied to the metabolites levels. Metabolite traits with > 20% missing were excluded and missing values were imputed to the day minimum. This produced 209 metabolites for the study. We used both plasma and serum samples as they are highly correlated and depend on the same genes [28]. However, we initially investigated possible differences between the metabolomic profiles derived from fasting serum and fasting plasma collected from the same twin volunteers. We have found that the results on plasma and serum were in keeping with one another and elected, therefore, to increase the sample size and hence the power by using both sample types. Moreover, we adjusted for the specimen type in statistical analysis (see Statistical analysis section). Throughout we refer to this plasma and serum metabolome as the “circulating metabolome”.

### 2.3 C-reactive protein

C-reactive protein (CRP) levels from serum of twin volunteers were established as described elsewhere [29]. The raw values of CRP were normalized by inverse transformation of ranks prior to statistical analysis.

### 2.4 Statistical analysis

The statistical analysis was carried in three steps. First, risk factors for fatigue were sought using univariate and multivariable mixed-effects regression including CWP, BMI and age, adjusting for family structure and zygosity. Then, we assessed associations between fatigue and metabolite levels using analysis of covariance (ANCOVA) by fitting linear mixed-effects models with metabolites as dependant variables and fatigue an independent factor, also adjusting for age, BMI, biological specimen type (plasma or serum) and processing batch, family structure and zygosity. Mediation analysis stratifying for presence/absence of CWP was carried out to determine if BMI was a mediator of the effects of fatigue on the levels of metabolites. Statistical significance threshold was set at p < 0.0005 corresponding to 100 independent tests with Bonferroni correction. The number of independent tests was estimated using the correlation structure of the observed metabolites in the TwinsUK dataset [30]. Finally, we assessed predictive capacity of the circulating metabolome for fatigue using ROC-analysis. Association between CRP levels and fatigue was assessed using ANCOVA in the same way as for the metabolites. Adjustment was made for age, BMI, family structure and zygosity. All analyses were performed in R using basic functions and packages “lme4” [31], “mediation” [32] and “PredictABEL” [33].

## 3 Results

### 3.1 The prevalence and risk factors for fatigue

A total sample of 4898 twins from the TwinsUK dataset has been assessed for fatigue using postal questionnaires. Complete covariate data were available for 2055 female individuals, which have been analysed further. In this sample, the prevalence of fatigue was 22.3% (Table 1) with much higher frequency in individuals with CWP as compared to those without CWP (38.6 vs 15.2%, p = 2.2 × 10^− 16^).

**Table 1:** Demographics of females from the TwinsUK dataset assessed for the presence of fatigue.

Subgroup	Prevalence of fatigue, %	Age (± SD), years	BMI (± SD), kg/m^2^
Total sample, n = 2055	22.3	54.4 ± 14.2	26.1 ± 5.2
With CWP, n = 621	38.6	59.6 ± 10.7	27.4 ± 5.6
Without CWP, n = 1434	15.2	52.2 ± 14.9	25.5 ± 4.9

CWP, chronic widespread pain.

Both in the CWP group and in non-CWP group, fatigue was associated with the increased BMI: 28.7 ± 6.4 vs 26.7 ± 4.9 kgm^− 2^ (p = 3.9 × 10^− 5^) in individuals with CWP and 26.2 ± 5.2 vs 25.4 ± 4.8 kgm^− 2^ (p = 0.030) in those without CWP, respectively. Also, females with fatigue were younger that those without it, though it was only marginally statistically significant in the non-CWP group: 57.7 ± 11.1 vs 60.8 ± 10.2 years (p = 5 × 10^− 4^) with CWP and 50.4 ± 15.5 vs 52.5 ± 14.8 years (p = 0.060) in those without CWP.

CWP was significantly associated with fatigue independent of BMI and age (Table 2) as it remained highly statistically significant with similar effect size after including these risk factors in the regression model. The effects of BMI and age were also statistically significant in a multivariable model including CWP, though their effects decreased suggesting some collinearity between CWP and age and BMI (Table 2).

**Table 2:** The effect of CWP on the risk of fatigue.

Model	Factor	Effect (β)	SE	p-Value
Univariate	CWP	1.288	0.112	6.0e–26
Multivariable	CWP	1.319	0.131	5.7e–24
	BMI	0.051	0.011	7.6e–6
	Age	− 0.016	0.005	6.5e–4

Logistic regression analysis of the dependence between fatigue and CWP adjusting for family structure and zygosity.

Mediation analysis showed that 6% of the total effect of CWP on the risk of fatigue is mediated via BMI (p = 0.0002), while the estimated mediation effect of CWP exceeded total and direct effects of BMI on the risk of fatigue, suggesting that the effect of BMI is not independent of the effect of CWP. The results show that CWP is a strong and independent risk factor for fatigue, while BMI is secondary to CWP as a risk factor of fatigue. Taking this into account, the subsequent analysis of association between the circulating metabolites and fatigue was carried out in groups stratified by the presence of CWP.

### 3.2 Association between fatigue and the circulating metabolome

**Fig. 1:**
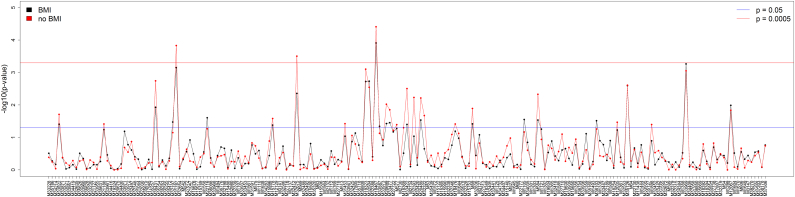
P-values (− log10) for the analysis of associations between fatigue and circulating metabolome. P-values are provided for linear mixed-effects models including and excluding BMI as a covariate.

A total of 209 circulating metabolites were tested for association with fatigue in 1106 twin females stratified by the presence of CWP (fatigue diagnosed in 114 out of 395 twins with CWP and in 93 out of 711 twins without CWP). In the non-CWP group, no statistically significant associations of fatigue and the metabolites was found after adjustment for multiple testing (Supplementary Table 2). In the CWP group, essential fatty acid eicosapentaenoate (EPA) was found to be associated with fatigue, being significantly decreased in cases (β = − 0.452 ± 0.116; p = 1.2 × 10^− 4^; Fig. 1; Supplementary Table 3).

Exclusion of BMI from the regression model showed the association with EPA to remain statistically significant with similar effect size (β = − 0.480 ± 0.115; p = 3.9 × 10^− 5^) and two other metabolites also reached statistical significance for association with fatigue: 3-carboxy-4-methyl-5-propyl-2-furanpropanoate lipid (CMPF), and C-glycosyltryptophan amino acid (β = − 0.406 ± 0.106; p = 1.5 × 10^− 4^ and β = 0.390 ± 0.107; p = 3.1 × 10^− 4^, respectively) (Fig. 1; Supplementary Table 3).

Mediation analysis for EPA, CMPF, and C-glycosyltryptophan was carried out based on the assumption that BMI may mediate the effects of fatigue. A statistically significant mediation effect of BMI was established for CMPF and C-glycosyltryptophan, but not for EPA (Table 3). Mediation effect relative to the total effect was 13% and 25% for CMPF and C-glycosyltryptophan, respectively.

**Table 3:** Direct and BMI-mediated effects of fatigue on circulating metabolite levels.

Metabolite	Effect of fatigue mediated via BMI (mediation effect)	Direct effect of fatigue accounting for mediation effect of BMI	Total effect (summary of mediated and direct effects)	Proportion of the total effect attributable to mediation effect
EPA	− 0.01 [− 0.03;0.008] p = 0.27	− 0.16 [− 0.26;− 0.07] p < 0.001	− 0.17 [− 0.27;− 0.08] p < 0.001	0.06 [− 0.05;0.24] p = 0.27
CMPF	− 0.02 [− 0.05;− 0.001] p = 0.04	− 0.15 [− 0.26;− 0.03] p = 0.01	− 0.17 [− 0.28;− 0.06] p < 0.001	0.13 [0.005;0.47] p = 0.05
C-glycosyltryptophan	0.05 [0.02;0.09] p < 0.001	0.15 [0.04;0.26] p = 0.01	0.20 [0.09;0.31] p < 0.001	0.25 [0.10;0.62] p < 0.001

Mediation analysis to reveal the effect of fatigue on EPA levels mediated via BMI.

### 3.3 Association between fatigue and serum C-reactive protein

To check if fatigue was associated with occult inflammation, we analysed serum levels of CRP in groups stratified by fatigue and CWP. Using ANCOVA with adjustment for covariates, the levels of CRP were found to be statistically significantly increased in females with fatigue in the CWP group, but not the non-CWP group (β = 0.189 ± 0.083; p = 0.023 and β = 0.133 ± 0.084; p = 0.112, respectively).

### 3.4 Predictive capacity of the circulating metabolome for fatigue

**Fig. 2:**
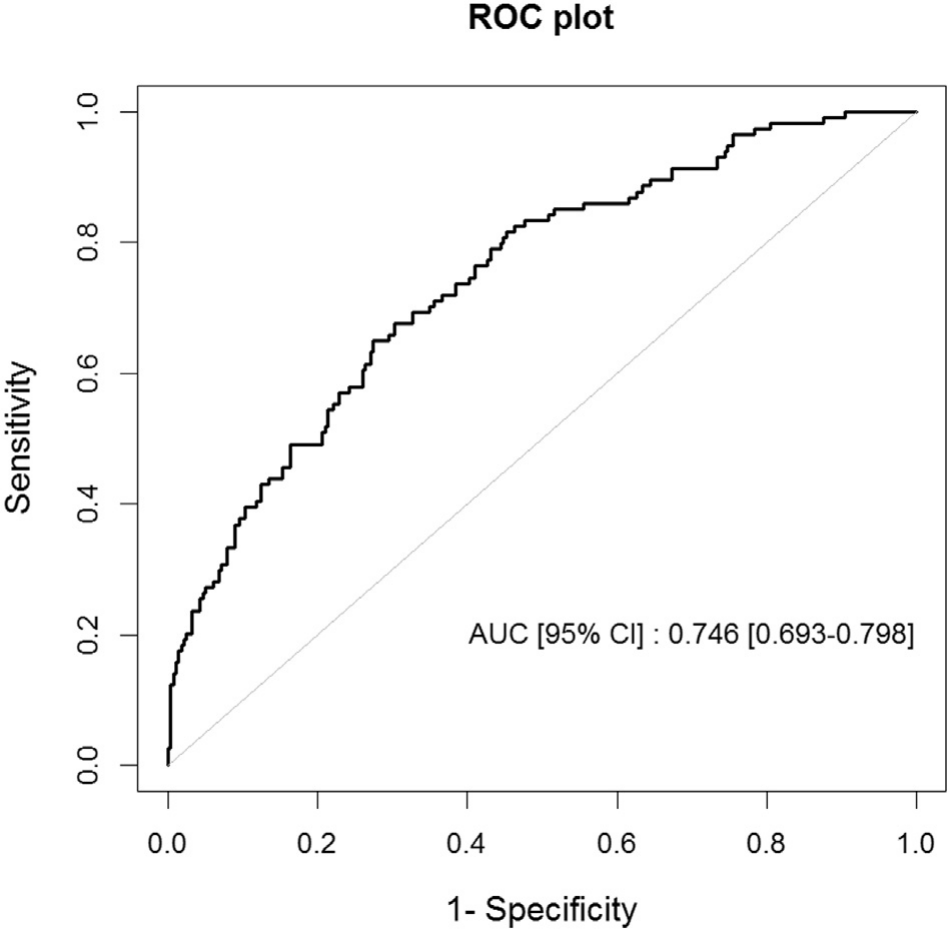
ROC plot for prediction of fatigue in CWP using 15 circulating metabolites. A combination of the metabolites was selected from all possible combinations (two, three, four, etc.) by choosing the best predictive model as detailed in the main text (Table 4).

Regression models were fitted with predictors comprising all possible combinations (pairs, threes, fours, etc.) of metabolites for which p-value was < 0.25 for association with fatigue in females with CWP. Best fit models for each combination sets were chosen based on Akaike Information Criterion followed by the ROC-analysis to check their predictive capacity for fatigue in CWP. The highest accuracy was achieved with a combination of 15 circulating metabolites, giving area under curve (AUC) = 0.746 [95% CI: 0.693–0.798], with the cross-validation error equal to 0.19 as established by jack-knife resampling (Table 4; Fig. 2). We additionally checked the performance of the model using twin pairs discordant for fatigue from the CWP group. Overall, there were 16 such the pairs in the dataset including 8 MZ and 8 DZ. Risks of fatigue were estimated using the predictive model and compared with known fatigue status of the twins. The prediction accuracies were 68.8% (95% CI 41.3%–89.0%, p = 0.105) for the MZ twins and 50.0% (28.2%–71.8%, p = 0.584) for the DZ twins.

**Table 4:** Circulating metabolites for the best predictive model for fatigue in CWP.

Metabolon_ID	Biochemical	Pathway	Sub-pathway	Level in fatigue
M18476	glycocholate	Lipid	Bile acid metabolism	Increased
M15500	carnitine	Lipid	Carnitine metabolism	Decreased
M01105	linoleate (18:2n6)	Lipid	Essential fatty acid	Decreased
M31787	3-carboxy-4-methyl-5-propyl-2-furanpropanoate (CMPF)	Lipid	Fatty acid/dicarboxylate	Decreased
M27722	erythrose	Carbohydrate	Fructose/mannose/galactose/starch/and sucrose metabolism	Increased
M20489	glucose	Carbohydrate	Glycolysis/gluconeogenesis/pyruvate metabolism	Increased
M00059	histidine	Amino acid	Histidine metabolism	Decreased
M12067	undecanoate (11:0)	Lipid	Medium chain fatty acid	Decreased
M21188	1-stearoylglycerol (1-monostearin)	Lipid	Monoacylglycerol	Increased
M32197	3-(4-hydroxyphenyl)lactate	Amino acid	Phenylalanine & tyrosine metabolism	Decreased
M35126	phenylacetylglutamine	Amino acid	Phenylalanine & tyrosine metabolism	Increased
M22130	phenyllactate (PLA)	Amino acid	Phenylalanine & tyrosine metabolism	Increased
M32675	C-glycosyltryptophan	Amino acid	Tryptophan metabolism	Increased
M35431	2-methylbutyroylcarnitine	Amino acid	Valine/leucine and isoleucine metabolism	Increased
M22116	4-methyl-2-oxopentanoate	Amino acid	Valine/leucine and isoleucine metabolism	Decreased

## 4 Discussion

This is the first population study of fatigue in CWP using metabolomics screening. We found fatigue associated with lower circulating levels of the eicosapentaenoate (EPA) ω-3 fatty acid in those with CWP. No association between EPA levels and CWP itself were seen either in the current study or in our earlier metabolome investigation of CWP alone [17]. This suggests that the decrease of EPA is a characteristic metabolic feature of CWP-associated fatigue and may serve as a biomarker for this condition. It further suggests that CWP and fatigue do not fully overlap metabolically and do, therefore, represent distinct physiological syndromes. Our results are concordant with previous findings of EPA level decrease in patients with ME/CFS that suggested that ω-3 fatty acid availability is linked to immune pathophysiology of the syndrome [34].

EPA is known to exhibit anti-inflammatory effects through a variety of mechanisms including decreased production of chemoattractants, down-regulation of NFκB, production of eicosanoids competing with pro-inflammatory molecules and other processes [35]. At the same time, inflammatory model of chronic fatigue is one of the most widely accepted; pro-inflammatory cytokines originating from systemic or peripheral inflammation act on the brain and initiate so called sickness behaviour characterised by loss of appetite, sleepiness, decreased social activity, depression and fatigue [36], [37]. Hypothetically, the decrease of EPA levels may result in the downregulation of anti-inflammatory pathways and promote the development of fatigue. This is additionally supported by the observed elevated serum levels of C-reactive protein in people reporting fatigue in the CWP group.

Given that the reduced EPA levels found in our study were unique to individuals with CWP and fatigue combined, it suggests there may be notable differences between fatigue in individuals with CWP in comparison to people who present with fatigue alone. Among the metabolites that were found at least nominally statistically significantly associated with fatigue in the non-CWP group, the top ones were amino-acids 2-hydroxyisobutyrate, glutamate, *N*-acetylthreonine, and 3-methylhistidine, and a peptide gamma-glutamylvaline (Supplementary Table 2). This suggests that fatigue reported by the non-CWP group may rather be related to muscle fatigue and wasting [38], and thus is intrinsically distinct from fatigue reported by the CWP group.

Apart from EPA, other essential fatty acids, such as docosapentaenoate (DPA) and docosahexaenoate (DHA) were decreased in fatigue in CWP group, though these did not reach the statistical significance threshold (Supplementary Table 3). All these metabolites occur in the body via dietary routes; either via direct consumption of ω-3 containing foods (e.g. fish or fish oils) or via the dietary precursor alpha-linolenic acid [39]. There are a number of reasons why low EPA levels may be seen in individuals at risk of fatigue in CWP. One of these may be significant dietary differences between those with CWP and controls. Individuals with CWP more likely to have a diet high in fat and low in fruit and vegetables [18]; this type of “Western” style diet has been found to be low in ω-3 fatty acids (reflected in low consumption of fish, fruit and vegetables) despite high amounts of saturated fats [40]. It is unknown as to why such dietary differences are found in individuals with CWP, although it can be hypothesised that it may be related to common comorbid factors such as a depressive mood state, which may lead to greater consumption of high fat foods which induce a pleasurable mood state during or shortly after consumption [41]. A reduction in dietary quality below some critical level could feasibly lead to further ill health due to a lack of nutrients, including low levels of EPA.

This raises a question of “healthy/normal” levels of EPA and other essential fatty acids. At present, despite a growing scientific literature devoted to supplementation in various states of ill health, the normal range of circulating EPA is yet to be agreed [42]. Given that TwinsUK is known to be representative of a general population [26], the levels of ω-3 fatty acids measured in our sample could serve as a normal baseline for Northern Europeans of a respective age group. Unfortunately, the Metabolon platform used in the current study do not allow absolute quantification of the metabolites; therefore, we cannot provide such values in our sample from TwinsUK. However, the absolute levels of ω-3 fatty acids were reported for about 900 generally healthy middle age and elderly women from the same TwinsUK resource using another platform: DHA 0.14 ± 0.05 mmol/L; EPA + DHA 0.44 ± 0.14 mmol/L [43].

In the CWP group, we also observed an association between fatigue and CMPF and C-glycosyltryptophan with a significant mediation effect of BMI. To the best of our knowledge, none of these metabolites are currently known to be related to chronic pain or fatigue, though C-glycosyltryptophan is associated with aging and age-related traits [27] and CMPF is related to chronic renal failure and renal cell damage [44]. Thus, these metabolites may be considered as new biomarkers of fatigue in CWP and their study may improve our understanding of the nature of this condition.

We identified a set of 15 circulating metabolites that may provide a relatively strong biomarker of fatigue in CWP (AUC 0.746 [95% CI: 0.693–0.798]). Taking advantage of powerful discordant twin-pairs design, the accuracy of the model was estimated to be 68.8% for MZ twins and 50.0% for DZ twins. These estimates were obtained in a very limited sample (8 pairs of twins in each group), therefore must be treated with caution. As fatigue associated with CWP may be a manifestation of CFS, using this biomarker tool may be able to provide a diagnostic of this syndrome and help differentiate CWP and CFS. A recent targeted metabolome study of CFS/ME produced a very accurate classifier with AUC estimates equal to 94%–96% [22]. There are several reasons for the lower accuracy of our panel. First, we did not deal with clinically proven diagnosis of ME/CFS, rather with the self-reported fatigue, thus resulting in a higher heterogeneity of our sample. Second, we contrasted fatigue vs non-fatigue in a specific context – among people with CWP – so the diagnostic accuracy may decrease as CWP itself is characterised by a specific metabolic profile [17] and shares pathogenetic links with fatigue [11].

To date, metabolomic studies of fatigue are limited to animal models and clinical samples. In patients with RA, increasing fatigue scores were associated with a metabolic pattern characterised by down-regulation of metabolites from the urea cycle, fatty acids, tocopherols, aromatic amino acids, and hypoxanthine [24]. Urine metabolome studies showed a significant correlation between aminohydroxy-*N*-methylpyrrolidine and beta-alanine with CFS expression [20], [21]. In a rat model of fatigue, distinctive changes in plasma metabolites related to branched-chain amino acid metabolism, urea cycle, and proline metabolism in the fatigued group were discovered [19]. The most recent clinical study of 612 plasma metabolites identified abnormalities in 20 metabolic pathways associated with CFS [22].

Our study has several limitations. We analysed only females, so results are not applicable to males. Another limitation is the use of questionnaires obtained over different years with various definitions of fatigue. However, there was no difference in fatigue prevalence between different definitions; also, the prevalence of fatigue in our sample, 22.3%, is close the figure of 20.9% obtained in a similar population study of Swedish twins [45]. Also, the association between EPA levels and fatigue was only established in participants with CWP, thus this finding cannot be generalized to fatigue associated with other conditions. The study of Swedish twins identified several sub-classes of fatigue each characterised by a combination of such symptoms as impairment, memory deterioration, sore throat, tender lymph nodes, muscle pain, multi-joint pain, headache, unrefreshing sleep, and post-exertional malaise [45]. This suggests that fatigue should be considered as a heterogeneous condition. We have not sub-classified fatigue in our study, but this would be a reasonable next step. Finally, given the cross-sectional design of the current study, all proposed mechanistic links remain speculative and require testing in a randomized controlled study or using instrumental analysis such as Mendelian randomization [46].

The following are the supplementary data related to this article.

Supplementary Table 1 Questionnaires used to define fatigue in the current study. Supplementary Table 1

Supplementary tables Image 1

## Conflict of interests

None to disclose.

## Funding

This work was funded by Arthritis Research UK grant #20682, the EU FP7 project Pain_Omics (contract #602736) and Chronic Disease Research Foundation grant on fibromyalgia. TwinsUK: the study was funded by the Wellcome Trust grant #202786/Z/16/Z; European Community's Seventh Framework Programme (FP7/2007-2013). The study also receives support from the National Institute for Health Research (NIHR)-funded BioResource, Clinical Research Facility and Biomedical Research Centre based at Guy's and St Thomas' NHS Foundation Trust in partnership with King's College London. CM is funded by the MRC AIM HY (MR/M016560/1) grant.

## Transparency document

Transparency document. Image 2
