# Supplementary material for: Metabolomic markers of fatigue: Association between circulating metabolome and fatigue in women with chronic widespread pain
Source: Biochim Biophys Acta. 2018 Feb;1864(2):601–6. doi: 10.1016/j.bbadis.2017.11.025 (PMC5764223; doi:10.1016/j.bbadis.2017.11.025)
Supplement: Supplementary Table 1 — Questionnaires used to define fatigue in the current study. [file mmc1.pdf]

Supplementary Table 1 – Questionnaires used to define fatigue in the current study

Questionnaire	Questions	Variant answers / Key	Fatigue defined for the current study
2000	Q4_25 "How often did you get fatigue (tiredness) in the last year?"	Does not occur or less than once a month / 0<br>Occurs about once a month / 1<br>Occurs about once a week / 2<br>Occurs daily / 3<br>Occurs several times a week / 4	Q4_25 ≥ 1 AND Q4_26 ≥ 2
	Q4_26 "How much did fatigue (tiredness) bother you in the last year?"	Not a problem / 0<br>It bothers me slightly / 1<br>It bothers me moderately / 2<br>It bothers me severely / 3<br>It bothers me extremely / 4	
2002	Q7_112 "Over the past three months, have you often felt tired or fatigued?"	No / 0<br>Yes / 1	Q7_112 = 1 AND Q7_113 = 1
	Q7_113 "Does tiredness or fatigue significantly limit your activities?"	No / 0<br>Yes / 1	
2008	Q14_87 "Over the past three months, have you often felt tired or fatigued?"	No / 0<br>Yes / 1	Q14_87 = 1 AND Q7_113 = 1
	Q14_88 "Does tiredness or fatigue significantly limit your activities?"	No / 0<br>Yes / 1	
